# Treating the patient and not just the cancer: therapeutic burden in prostate cancer

**DOI:** 10.1038/s41391-021-00328-1

**Published:** 2021-02-18

**Authors:** Daniel E. Spratt, Neal Shore, Oliver Sartor, Dana Rathkopf, Kara Olivier

**Affiliations:** 1 Michigan Medicine, Ann Arbor, MI USA; 2 Carolina Urologic Research Center, Atlantic Urology Clinics, Myrtle Beach, SC USA; 3 Tulane University School of Medicine, New Orleans, LA USA; 4 Memorial Sloan Kettering Cancer Center, New York, NY USA; 5 Massachusetts General Hospital Cancer Center, Boston, MA USA

**Keywords:** Cancer therapy, Prostate cancer

## Abstract

**Background:**

Prostate cancer (PC) is a leading cause of death in older men. Androgen deprivation therapy (ADT) is considered the standard-of-care for men with locally advanced disease. However, continuous androgen ablation is associated with acute and long-term adverse effects and most patients will eventually develop castration-resistant PC (CRPC). The recent approval of three, second-generation androgen receptor inhibitors (ARIs), apalutamide, enzalutamide, and darolutamide, has transformed the treatment landscape of PC. Treatment with these second-generation ARIs have produced positive trends in metastasis-free survival, progression-free survival, and overall survival. For patients with non-metastatic CRPC, who are mainly asymptomatic from their disease, maintaining quality of life is a major objective when prescribing therapy. Polypharmacy for age-related comorbidities also is common in this population and may increase the potential for drug–drug interactions (DDIs).

**Method:**

This review summarizes the multiple factors that may contribute to the therapeutic burden of patients with CRPC, including the interplay between age, comorbidities, concomitant medications, the use of ARIs, and financial distress.

**Conclusions:**

As the treatment landscape in PC continues to rapidly evolve, consideration must be given to the balance between therapeutic benefits and potential treatment-emergent adverse events that may be further complicated by DDIs with concomitant medications. Patient-centered communication is a crucial aspect of alleviating this burden, and healthcare professionals (HCPs) may benefit from training in effective patient communication. HCPs should closely and frequently monitor patient treatment responses, in order to better understand symptom onset and exacerbation. Patients also should be encouraged to participate in exercise programs, and health information and support groups, which may assist them in preventing or mitigating certain determinants of the therapeutic burden associated with PC and its management.

## Introduction

Worldwide, older populations continue to grow at an unprecedented rate. By 2030, it is projected that nearly 12% of the global population will be aged 65 years or older [1]. Conventionally, a person in this age range is referred to as “elderly” [2]. However, chronology alone may not reliably predict the rate of physiological decline owing to environmental factors and comorbidities. Differences in genetics, lifestyle, and overall health status suggest a heterogeneity to the aging process. It is important to consider such diversity, particularly with respect to the individualization of treatment regimens [3].

Prostate cancer (PC) develops predominantly in older patients with an average age at diagnosis of 66 years [4]. Older patients may be more likely to experience aggressive forms of the disease compared with their younger counterparts. However, studies of the subset of men aged <50 years diagnosed with advanced PC provide strong evidence of poor survival in this younger age group [5]. The association between age and PC aggressiveness has been confirmed in several prospective and retrospective studies [6–8]. PC is the fifth leading cause of cancer-related death in men worldwide [9], with an incidence rate of ~60% in men aged older than 65 years [10] and an estimated 33,330 deaths in 2020, representing ~10% of all male deaths [11].

Androgen deprivation therapy (ADT) is the standard-of-care for patients with advanced PC [12]. Although proven to be efficacious, ADT is associated with acute and long-term drug-related adverse effects. Many patients with rising prostate-specific antigen (PSA) levels eventually progress to castration-resistant PC (CRPC), often associated with persistent androgen receptor (AR) signaling, despite maintaining castrate concentrations of serum testosterone, defined as <50 ng/dL [13, 14].

This review aims to discuss the potential clinical impact of the interplay between age, comorbidities, concomitant medications, and second-generation androgen receptor inhibitors (ARIs) on the therapeutic burden of patients with CRPC. In the context of this review, we define therapeutic burden as the clinical impact on patients from the intersecting factors of adverse events (AEs), drug–drug interactions (DDIs), chronic comorbidities, polypharmacy, financial burden, and the involvement of multiple healthcare professionals (HCPs) in patient management.

## Factors defining therapeutic burden

Therapeutic burden is typically defined as the workload of healthcare and its impact on patient functioning and wellbeing. The “workload” includes the demands on patient time and energy owing to treatment, including understanding prescribed or administered therapies, attending appointments, arranging supportive services, and adhering to a medication schedule. The “impact” on patient function includes the effect of the workload on the behavioral, cognitive, physical, and psychosocial wellbeing of the patient [15, 16].

The interdependent components that contribute to therapeutic burden include polypharmacy, AEs, chronic comorbidities, long-term concomitant medications, and suboptimal communication between patients and HCPs [16]. Increased therapeutic burden is associated with poor treatment compliance, wasted resources, and suboptimal clinical outcomes [17]. Therefore, these factors should be considered when adding ARIs to ongoing ADT and patient-centric treatment selections should be prioritized whenever possible.

### Communication between patients and HCPs

Barriers to communication between patients and HCPs might also contribute to the therapeutic burden of patients with PC. For example, patients may believe that they cannot effectively describe their symptoms as they lack the appropriate vocabulary. Building a patient-focused relationship based on individual and family needs strengthens trust and mutual engagement throughout the cancer journey.

Patient-centered communication is particularly important when developing a care plan: allaying fears, managing pain, and adverse treatment effects, as well as the timely referral to hospice for end-of-life care [18]. Patient-centered communication with HCPs has been associated with better health-related quality of life (HRQoL) [19], emphasizing the importance of open, bidirectional dialog.

Insufficient patient-centered information on treatment, access to, and coordination of care are among the most notable perceived deficiencies in patient experience with cancer care [20, 21]. Studies have found that clinicians request patient preferences in medical decisions only ~50% of the time [22, 23]. Furthermore, patients who felt more involved in the decision-making process were likely to feel more confident in their decisions [23].

Communication between the HCP and the primary caregiver is equally valuable. Partners or caregivers contribute importantly to disease management and are major providers of emotional support, which may adversely affect their own health and quality of life (QoL) [24]. Partners of patients with PC also report greater degrees of emotional distress than the patients themselves, as personal health issues increase, with a prevalence of anxiety and depression reported in ~47% and 42%, respectively [24–26]. Coherent communication with HCPs could help identify positive coping strategies for the primary caregiver and possible interventions to facilitate communication among couples navigating PC treatment [25].

Clinicians may lack sufficient formal training in communication, which affects recognition of and response to the informational and emotional needs of the patient [18]. In addition, opportunities exist to better harmonize communication among HCPs by aligning electronic healthcare records across interdisciplinary care [27].

### Financial burden of PC

“Financial toxicity” is becoming a familiar term used in the discussion of cancer therapies. Defined as the objective financial burden and subjective financial distress of patients with cancer, as a result of innovative drug treatment and concomitant health services, financial distress for patients with PC is not isolated from the overall anxiety and discomfort experienced as a result of a cancer diagnosis and its ensuing therapeutic interventions [28].

Financial toxicity has been described as a corollary to cancer treatment akin to nausea and hair loss [28]. The combination of emotional distress and financial obligations associated with cancer care may impede the ability of the patient to cope effectively with the disease, its symptoms and its treatment thereby adversely affecting health outcomes. In the context of patient-centered cancer care, the clinician also can assist in containing the financial burden and distress to an individual patient with cancer. In 2017, The American Cancer Society developed a list of questions on cost-of-care that patients diagnosed with cancer might wish to pose to their HCP and which might facilitate the urgent dialog on avoiding low-value treatment while pursuing concrete solutions to preserve financial health [28].

The application of validated instruments, such as the COmprehensive Score for financial Toxicity (COST) measure, to sensitive discussions of treatment affordability with the patient, may assist the clinician to better explore and appreciate individual spending habits, financial resources and, psychosocial responses of the patient with cancer to financial burden [29]. Other commonly employed, multidimensional HRQoL instruments fail to capture the “social impact” of serious disease, treatment, and financial variables. The early literature demonstrates that issues can span the range of employment, activities of daily living, and finances while later works continue to expose the social issues of significant concern for patients with cancer. Both qualitative research and economic evaluations have illustrated that financial worry and hardship are both evident and prevalent in this patient population [30].

The initial treatment and subsequent monitoring of patients with PC places a heavy burden on the US healthcare system. The average per-patient lifetime cost of PC treatment averages ~$34,000 and PC-related costs represent up to one-third of total medical care depletion [31]. Studies also have indicated a stage effect of PC on the cost of treatment, escalating incrementally as patient risk increases. The aging population of patients with PC will augment the clinical and economic burden of PC on the healthcare system and, therefore, the choice of initial treatment could potentially limit healthcare expenditures and resources [32].

### Comorbid conditions

Chronic comorbid conditions commonly occur among older men with PC, with >50% having at least one pre-existing chronic condition, the most prevalent being cardiometabolic and chronic respiratory diseases or disorders [27, 33].

In those patients who live with numerous, non-malignant comorbid conditions, increased drug burden often can be associated with diminished physical function, attention, concentration, and medication non-adherence [27]. Furthermore, comorbidity patterns involving cardiovascular, metabolic diseases, mental illness, and musculoskeletal disorders have been linked to diminished HRQoL [34].

When electing to add an ARI to ongoing ADT, HCPs should base their selection on the approved indication, proven efficacy, drug safety and tolerability, life expectancy with respect to curative intent, accessibility to medication, cost-of-drug, patient preference, HRQoL, and available psychosocial support, as well as the more global effects of DDIs associated with polypharmacy [35, 36]. Cross comparisons of comorbidity impact on survival outcomes in patients with PC is challenged by the heterogeneity of study designs and assessment techniques [37]. A standardized, patient-centric, quantitative measure of multimorbidity, that may be applied readily in both research and clinical practice is needed, to better characterize the growing population of patients with comorbid conditions [38].

## AR-targeted therapeutic options for PC

Biochemical recurrence is experienced by 27–53% of patients who have previously undergone either prostatectomy or radiotherapy [12]. Resistance mechanisms to ADT treatment have been associated primarily with aberrant AR signaling [39]. These mechanisms contribute to neoplastic cellular proliferation and tumor progression to CRPC [14, 40].

CRPC is defined by a rising PSA concentration and/or radiographical progression despite a castrate serum testosterone level <50 ng/dL. In the castration-resistant patient, metastases detected by conventional imaging with computerized tomography or technetium-99m scintigraphy is defined as metastatic CRPC (mCRPC), whereas CRPC without radiographic evidence of metastases is categorized as non-metastatic CRPC (nmCRPC) [12, 13].

Since 2012, the treatment landscape for CRPC in the US has been transformed following the approval of abiraterone acetate/prednisone (approved for mCRPC) [41] and the second-generation ARIs [42] enzalutamide (approved for mCRPC and nmCRPC), apalutamide (approved for nmCRPC), and darolutamide (approved for nmCRPC) [43–45]. Ongoing and future clinical trials are expected to broaden the indications for ARIs to earlier stages of PC, possibly including the neoadjuvant setting [46, 47].

Although ADT is generally well tolerated, androgen depletion may exert negative effects on cognitive function, cardiovascular health, sexual health, insulin sensitivity, and bone health (including falls and fracture) [48, 49]. Therefore, clinicians should consider how ARI-related toxicities may affect patients with PC and chronic comorbidities managed with long-term concomitant medication, and how multiple interdependent factors might contribute to the therapeutic burden of CRPC.

### Clinical profiles of second-generation ARIs for CRPC

Enzalutamide, apalutamide, and darolutamide were approved based on significant improvements in both overall survival (OS) and metastasis-free survival (MFS) versus placebo in phase 3 clinical trials (Table 1).

**Table 1:** Efficacy and safety outcomes in recent clinical trials in patients with CRPC.

Trial	Description	Population	Primary trial outcomes (all drug vs PBO)	Secondary trial outcomes (all drug vs PBO)	Discontinuation rates^†^ (drug vs PBO)	Adverse events, occurring in >10% of patients in treatment arm (all drugs vs PBO)
*Enzalutamide*						
PROSPER [50, 51] NCT02003924	Randomized study to examine the safety and efficacy of enzalutamide in patients with nmCRPC (*n* = 1401) Enzalutamide dose: 160 mg once daily	nmCRPC BL PSA ≥ 2 ng/mL PSADT ≤ 10 months ECOG PS 0–1 No lesions detectable by CT/MRI and bone scan No history of seizure	MFS assessed from randomization until radiographic progression at any time, or death 36.6 vs 14.7 months HR 0.29; 95% CI 0.24–0.35; *P* < 0.001	Primary analysis Median time to PSA progression: 37.2 vs 3.9 months HR 0.07; 95% CI 0.05–0.08; *P* < 0.001 Median time to first use of antineoplastic therapy: 39.6 vs 17.7 months HR 0.21; 95% CI 0.17–0.26; *P* < 0.001 Median OS: NR vs NR HR 0.80; 95% CI 0.58–1.09; *P* = 0.15 Final analysis Median OS: 67.0 vs 56.3 months HR 0.73; 95% CI 0.61–0.89; *P* = 0.001	9% vs 6%	Fatigue: 33% vs 14% Hot flashes: 13% vs 8% Nausea: 11% vs 9% Diarrhea: 10% vs 10% Hypertension^‡^: 12% vs 5% Fall: 11% vs 4% Dizziness: 10% vs 4% Decreased appetite: 10% vs 4%
PREVAIL [52] NCT01212991	Randomized study to examine the effect of enzalutamide on OS and PFS in patients with mCRPC (*n* = 1717) who progressed on ADT but did not receive chemotherapy Enzalutamide dose: 160 mg once daily	mCRPC ECOG PS 0–1 No prior cytotoxic chemotherapy No known brain metastasis Continued ADT	OS assessed from randomization to death due to any cause 32.4 vs 30.2 months HR 0.71; 95% CI 0.60–0.84; *P* < 0.001 PFS assessed from randomization to the first evidence of radiographic disease progression or death due to any cause NR vs 3.9 months HR 0.19; 95% CI 0.15–0.23; *P* < 0.001	Median time to PSA progression: 11.2 vs 2.8 months HR 0.17; 95% CI 0.15–0.20; *P* < 0.001 Median time to first use of cytotoxic chemotherapy: 28.0 vs 10.8 months HR 0:35; 95% CI 0.30–0.40; *P* < 0.001 Median time to first skeletal-related event: 31.1 vs 31.3 months HR 0.72; 95% CI 0.61–0.84; *P* < 0.001	6% vs 6%	Fatigue: 36% vs 26% Back pain: 27% vs 22% Constipation: 22% vs 17% Arthralgia: 20% vs 16% Decreased appetite: 18% vs 16% Hot flashes: 18% vs 8% Diarrhea 16% vs 14% Hypertension: 13% vs 4% Asthenia: 13% vs 8% Falls: 12% vs 5% Weight loss: 11% vs 8% Edema peripheral: 11% vs 8% Headache: 10% vs 7%
AFFIRM [53] NCT00974311	Randomized study to examine efficacy and safety of enzalutamide on OS in patients with mCRPC (*n* = 1199) Enzalutamide dose: 160 mg once daily	mCRPC ECOG PS 0–2 No known brain metastases	OS assessed from randomization to death due to any cause 18.4 vs 13.6 months HR 0.63 (95% CI 0.53–0.75) *P* < 0.001	Median radiographic PFS: 8.3 vs 3.0 months HR 0.40; 95% 0.35–0.47; *P* < 0.001 Median time to first skeletal event: 16.7 vs 13.3 months HR 0.69; 95% CI 0.57–0.84; *P* < 0.001 Median time to PSA progression: 8.3 vs 2.9 months HR 0.25; 95% CI 0.20–0.30; *P* < 0.001	8% vs 10%	Fatigue: 34% vs 29% Diarrhea: 21% vs 18% Hot flashes: 20% vs 10% Musculoskeletal pain: 14% vs 10% Headache: 12% vs 6%
*Apalutamide*						
SPARTAN [54, 55, 89, 93] NCT01946204	Randomized study to examine the safety and efficacy of apalutamide in patients with nmCRPC (*n* = 1207) Apalutamide dose: 240 mg once daily	nmCRPC PSADT ≤ 10 months No lesions detectable by CT/MRI or bone scan No history of seizure	MFS assessed from randomization to the first detection of distant metastasis or death by any cause 40.5 months with apalutamide vs 16.2 months with PBO HR 0.28; 95% CI 0.23–0.35; *P* < 0.001	Primary analysis Median time to metastasis; 40.5 vs 16.6 months HR 0.27; 95% CI 0.22–0.34; *P* < 0.001 Median PFS: 40.5 vs 14.7 months HR 0.29; 95% 0.24–0.36; *P* < 0.001 Median time to symptomatic progression: NR vs NR HR 0.45; 95% CI 0.32–0.63; *P* < 0.001 Median OS: NR vs 39.0 months HR 0.70; 95% CI 0.47–1.04; *P* = 0.07 Median time to the initiation of cytotoxic chemotherapy: NR vs NR HR 0.44; 95% CI 0.29–0.66 Final analysis Median OS: 73.9 vs 59.9 months HR 0.784; *P* = 0.0161 Median time to cytotoxic chemotherapy: NR vs NR HR 0.629; *P* = 0.0002	10.6% vs 7.0%	Fatigue^§^: 30% vs 21% Hypertension: 25% vs 20% Rash^§^: 24% vs 6% Diarrhea: 20% vs 15% Nausea: 18% vs 16% Weight loss: 16% vs 6% Arthralgia: 16% vs 8% Falls^§^: 16% vs 9% Fracture: 12% vs 7%
*Darolutamide*						
ARAMIS [56, 57, 94] NCT02200614	Randomized phase 3 study to examine the safety and efficacy of darolutamide in patients with nmCRPC (*n* = 1509) Darolutamide dose: 600 mg twice daily	nmCRPC BL PSA ≥ 2 ng/mL PSADT ≤ 10 months ECOG PS 0–1	MFS assessed from randomization to confirmed detection of distant metastasis or death from any cause 40.4 vs 18.4 months; HR 0.41; 95% CI 0.34–0.50; *P* < 0.001	Primary analysis Median OS: NR vs NR HR 0.71; 95% CI 0.50–0.99; *P* < 0.04 Median time to pain progression: 40.3 vs 25.4 months HR 0.65; 95% CI 0.53–0.79; *P* < 0.001 Median time to cytotoxic chemotherapy: NR vs 38.2 months HR 0.43; 95% CI 0.31–0.60; *P* < 0.001 Median time to first symptomatic skeletal event: NR vs NR HR 0.43; 95% CI 0.22–0.84; *P* < 0.01 Median PFS: 36.8 vs 14.8 months	8.9% vs 8.7%	Fatigue^||^: 12.1% vs 8.7%
				HR: 0.38; 95% CI 0.32–0.45 Median time to PSA progression: 33.2 vs 7.3 months HR 0.13; 95% CI 0.11–0.16 Final analysis Median OS: NR vs NR HR 0.69; 95% CI 0.53–0.88; *P* = 0.003 Median time to pain progression: 40.3 vs 25.4 months HR 0.65; 95% CI 0.53–0.79; *P* < 0.001 Median time to first cytotoxic chemotherapy: NR vs NR HR 0.58; 95% CI 0.44–0.76; *P* < 0.001 Median time to first symptomatic skeletal event: NR vs NR HR 0.48; 95% CI 0.29–0.82; *P* = 0.005		

^†^Discontinuation due to adverse events.

^‡^This adverse event includes increased blood pressure.

^§^The incidences of the following adverse events in the apalutamide group versus the placebo group were adjusted for exposure (events per 100 patient-years): fatigue (incidence, 32.3 vs 27.2), hypertension (36.3 vs 38.7), rash (29.6 vs 8.3), diarrhea (21.6 vs 22.6), nausea (15.8 vs 20.4), weight loss (18.3 vs 10.5), arthralgia (14.7 vs 8.0), falls (13.6 vs 10.0), fracture (10.5 vs 7.8), dizziness (7.7 vs 6.6), hypothyroidism (7.6 vs 2.2), mental impairment disorder (3.9 vs 3.4), and seizure (0.2 vs 0) [54].

^||^This category combines the following Medical Dictionary for Regulatory Activities, version 20.0 (MedDRA) terms: asthenic conditions, disturbances in consciousness, decreased strength and energy, malaise, lethargy, asthenia, and fatigue [56].

*ADT* androgen deprivation therapy, *CI* confidence interval, *CRPC* castration-resistant prostate cancer, *CT* computerized tomography, *ECOG* *PS* Eastern Cooperative Oncology Group Performance Status, *HR* hazard ratio, *mCRPC* metastatic castration-resistant prostate cancer, *MFS* metastasis-free survival, *MRI* magnetic resonance imaging, *nmCRPC* non-metastatic castration-resistant prostate cancer, *NR* not reached, *OS* overall survival, *PBO* placebo, *PFS* progression-free survival, *PSA* prostate-specific antigen, *PSADT* prostate-specific antigen doubling time.

#### Enzalutamide

In the PROSPER trial of patients with nmCRPC, enzalutamide plus ongoing ADT demonstrated significantly improved median MFS, reduced risk of radiographic progression or death, prolonged times to first use of new antineoplastic therapy, and PSA progression compared with placebo plus ADT [50]. In the final analysis of PROSPER, enzalutamide also was associated with significantly prolonged OS [51]. In chemotherapy-naive patients with mCRPC in PREVAIL, treatment with enzalutamide was associated with significantly improved OS compared with placebo [52]. In the AFFIRM trial, enzalutamide also was associated with significantly prolonged post-chemotherapy OS versus placebo in patients with mCRPC [53].

#### Apalutamide

In the SPARTAN trial, apalutamide plus ADT was associated with significant improvements in median MFS, progression-free survival (PFS), time to metastasis, and time to symptomatic progression compared with placebo plus ADT in patients with nmCRPC [54]. Apalutamide also was associated with significantly prolonged OS in the final analysis of the SPARTAN trial [55].

#### Darolutamide

In the ARAMIS trial, darolutamide plus ADT was associated with significantly longer median MFS compared with placebo plus ADT in patients with nmCRPC. Darolutamide also was associated with improvements in the secondary endpoints including OS, time to pain progression, time to cytotoxic chemotherapy, and time to a symptomatic skeletal event [56, 57]. In the final analysis of ARAMIS, darolutamide was associated with a significant improvement in OS compared with placebo, although median OS was not reached in either treatment group [57].

### Safety profiles

Maintained QoL and physical wellbeing are important considerations when treating patients with CRPC. Given the negative impact that treatment-emergent AEs can impose on QoL and their association with increased healthcare utilization and cost, therapeutic efficacy and response must always be balanced against the potential risk of amplifying treatment burden by impairing functional capacity and exacerbating comorbid symptoms [58]. Evidence from several clinical trials has confirmed an increased incidence of certain AEs associated with apalutamide or enzalutamide added to ADT versus placebo plus ADT (Table 1).

#### Enzalutamide

In PROSPER, the AEs associated with enzalutamide at an incidence of >10% compared with placebo included fatigue, hot flashes, nausea, falls, dizziness, decreased appetite, major cardiovascular events, and hypertension [50]. Similarly, in AFFIRM, a higher incidence of all grades of fatigue, diarrhea, hot flashes, musculoskeletal pain, and headache was reported with enzalutamide compared with placebo [53]. PREVAIL reported a higher incidence of falls in the overall population (12 vs 5%), as well as in the subgroup of patients aged ≥75 years (19 vs 8%) with enzalutamide versus placebo, respectively [52, 59].

In clinical trials, increased risk of seizure with enzalutamide has been observed in association with higher than recommended daily doses of 160 mg, or with concomitant medications or conditions [53, 60], which suggests the potential for enzalutamide to affect the central nervous system (CNS) [61].

#### Apalutamide

In SPARTAN, AEs associated with apalutamide that occurred at an incidence ≥15% compared with placebo included fatigue, rash, falls, mental impairment, and hypothyroidism [54].

#### Darolutamide

As darolutamide has only recently been approved, its published AE profile is limited to the pivotal ARAMIS phase 3 trial. The available data indicate that the safety profile of darolutamide reflects minimal increases in AEs of any grade beyond that of background ADT, with the exception of fatigue which was reported more frequently with darolutamide than with placebo [56]. No differences were reported between the two treatment arms in the incidence of falls, cognitive disorder, hypertension, and hypothyroidism [56]. In the final analysis of the ARAMIS trial, darolutamide was associated with minimal or no difference in the incidence of most ARI-related AEs, including falls, fractures, hypertension, rash, and mental impairment. Fatigue was the only AE with an incidence higher than 10% observed with darolutamide [57].

Darolutamide demonstrated low blood–brain barrier penetration in rodents, supported by a human neuroimaging study, which may be associated with a reduced risk of CNS AEs [56, 62–65]. Given that darolutamide was approved in 2019 for the treatment of nmCRPC, these results require confirmation with expanded safety data.

#### Managing ARI-related AEs

Aging itself may be associated with an increased fear of falling, which can promote restricted physical activity [66]. Exacerbated by a higher incidence of chronic comorbidities and long-term use of concomitant medication, immobility can further jeopardize physical function [67]. Thus, multiple, additive factors contribute to the therapeutic burden in the aging population of patients with PC.

Cancer-related fatigue is described as a “distressing” and “persistent” level of exhaustion related to cancer or cancer treatment that is not proportional to recent activity and interferes with usual functioning [68]. Patients experiencing fatigue have reported impaired ability to exercise, participate in productive employment, socialize, and perform daily living activities [68]. Regular exercise improves strength, maintains mobility, and balance, reduces the risk of falls, and attenuates symptoms of fatigue. Clinicians should encourage their patients to perform any amount or type of exercise at tolerable levels of intensity and/or duration [69–71].

Impaired cognitive function in patients with PC can complicate disease management and treatment decision-making, while impacting QoL and daily functioning. HCPs should be mindful of any pre-existing comorbidities, that may predispose the patient to further cognitive decline [72]. Furthermore, participating in group sessions that focus on improving memory skills, psychoeducation, and cognitive exercises can alleviate perceived symptoms of cognitive impairment that contribute to therapeutic burden and improve objective measures of attention and memory [73, 74].

Results from observational studies suggest that adherence to PC-specific diets may be associated with a decreased risk of disease progression [75]. Although patients have indicated that they would value dietary advice following a diagnosis of PC, HCPs have indicated that they do not routinely provide nutritional guidance as they may lack confidence in the dietary management of their patients with PC. HCPs should discuss aspects of nutrition and daily diet in the context of personalized disease management, notably in patients with comorbid conditions such as metabolic syndrome factors, diabetes, and obesity, which may adversely impact disease progression [75, 76].

### Potential for DDIs with second-generation ARIs

As many patients with PC are older and may have comorbidities that require long-term, concomitant medication, it is important to consider the potential for DDIs between second-generation ARIs and comedications used in this population [35, 77]. Common comorbidities in patients with PC include hypercholesterolemia, hypertension, diabetes mellitus, asthma, urologic complications, depression, and neurologic disorders [78], with frequently reported comedications including antihypertensives and agents for other cardiovascular disorders, analgesics, treatments for urological and gastrointestinal disorders, antidepressants, anxiolytics, and anti-dementia drugs [79].

Both apalutamide and enzalutamide exhibit the potential for DDIs when co-administered with medications that are substrates for several metabolizing enzymes and drug transporters. Apalutamide and enzalutamide have been linked to the induction of cytochrome P450 (CYP) 3A4, 2C9, and 2C19 [80, 81]. These enzymes metabolize up to 50% of medications [82]. In a post hoc analysis of the SPARTAN trial, the risk of falls was increased in patients receiving apalutamide and concomitant alpha-blockers or antidepressants [83]. Apalutamide also has the potential to interact with uridine-diphosphate glucuronosyltransferase, P-glycoprotein (P-gp), breast cancer resistance protein (BCRP), and organic anion-transporting polypeptide (OATP) 1B1 [81], whereas enzalutamide might also interact with CYP2C8 inhibitors or inducers [80]. Consequently, in order to reduce the risk of DDIs, the respective prescribing information recommends that practitioners avoid simultaneous use of apalutamide or enzalutamide with these medications [80, 81]. Medicinal products that should be used with caution include antithrombotics, such as dabigatran; antihypertensives, such as amlodipine, statins such as rosuvastatin, and cardiac glycosides, such as digoxin [81].

Darolutamide has a molecular structure distinct from that of apalutamide and enzalutamide [65], and in its pivotal phase 3 trial only demonstrated a potential to interact with BCRP substrates and combined P-gp and CYP3A4 inducers or inhibitors [44, 79]. In preclinical and phase 1 studies, interaction of darolutamide with rosuvastatin, a substrate for the drug transporters BCRP and OATP, was the only clinically relevant interaction, which did not appear to translate into increased AEs in an analysis of safety data from the ARAMIS clinical trial [79, 84].

The risk of DDIs is an important consideration when selecting an AR-targeted therapy for patients with CRPC at risk for high therapeutic burden, and while DDIs may not translate to a clinically significant increase in treatment-related AEs, patients should continue to be monitored carefully for any increased risk of potential toxicities induced by these interactions. The loss of efficacy when co-administered with long-term medications or increased risk of drug-related adverse effects also represent clinically relevant outcomes of DDIs.

## Validated instruments for assessment of QoL

Several validated assessment instruments and questionnaires have been developed to evaluate the impact of both disease and its treatment on physical and cognitive function in patients with PC. The choice of instrument may vary depending on whether the clinician intends to use it for screening symptoms or clinical research, and with which identifiable complaints the patient presents, e.g., the Functional Assessment of Cancer Therapy (FACT)-Cognitive tool explores different domains than the FACT-Prostate (FACT-P) tool (perceived cognitive function vs HRQoL). As various comorbidities and common complaints reported by patients with PC, e.g., fatigue and pain, may impact cognitive function, comprehensive assessment usually entails an evaluation of these symptoms. A lack of consensus prevails on the appropriate assessment instruments to apply to patients undergoing treatment for PC, particularly with respect to cognitive function [85, 86]. Table 2 details the distinguishing features of some commonly used assessment tools.

**Table 2:** Assessment tools.

Name of test	Domain(s) assessed	Tool structure	Other information
Brief Pain Inventory-Short Form [95]	Pain	9-item questionnaire. Each item is rated from 0, no pain, to 10, pain as bad as you can imagine, and contributes with the same weight to the final score	Captures other aspects of pain assessment (site of pain and pain treatment or medication Requires ~5 min to complete
Functional Assessment of Cancer Therapy-Cognitive Function [96, 97]	Perceived cognitive function	50-item questionnaire using four scales (perceived cognitive ability, perceived QoL impairment, perceived cognitive dysfunction, and comments from others)	Specific for cancer patients Current version 3 (at time of writing) Validated in patients with cancer Employs both negative and positive wording FACIT recommendation is to use 33 items and score the four subscales separately
Functional Assessment of Cancer Therapy-Fatigue [98]	Fatigue	13-item fatigue-specific test scale as a subscale adjunct to the 28-item FACT-G (fatigue rated over 7 days)	Designed/validated for cancer patients with anemia Brief (~10 min) and easy to score No specific cognitive measures
Functional Assessment of Cancer Therapy-Prostate [99]	Health-related QoL (prostate-specific)	12-item prostate cancer-specific test scale as a subscale adjunct to the general 27-item FACT-G health-related QoL questionnaire	Self-administered; requires between 10–14 min to complete Validated for QoL evaluation in men with PC
Cancer Fatigue Scale [100]	Fatigue	15-item scale with three subscales (physical, affective, cognitive)	Specifically designed to assess fatigue in patients with cancer; validated by correlation with visual analog scale
Patient Health Questionnaire [101]	Depression	9-item screening assessment measuring level of loss of interest, feelings of depression, loss of energy, sleep problems, and concentration problems	May also highlight issues with sleep and fatigue as well as aspects of cognitive functioning Normally used in original 1-dimensional form though a 2-dimensional form has been used in patients with prostate cancer
Functional Activities Questionnaire [102, 103]	Daily functioning (i.e., financial, organizational, social)	10 categories assessing activities of daily living (e.g., bill paying, shopping, cooking, traveling, remembering, current events)	Standardized scale Commonly used; high sensitivity/specificity Administered by healthcare professional to a patient or surrogate
European Organisation for Research and Treatment of Cancer Quality of Life Questionnaire C30 [99]	General health status and QoL	30-item cancer-specific measure of health status and health-related QoL; five functional domain scales (physical, role, emotional, social, cognitive), three symptom scales (fatigue, pain, emesis), global health status/QoL scale, and six further-symptom items	Current version 3 (at time of writing)
Short-form 36 health survey [99]	General health	36-item, 8-dimension scale (physical functioning, social functioning, role limitations due to personal problems, mental health, energy/vitality, role limitations due to emotional problems, pain, general health perception)	Self-administered by healthcare professional interview Generic health test No cognitive component
EQ-5D [99, 104]	General health	5-domain scale (mobility, self-care, usual function status, pain/discomfort, anxiety/depression) Intended for disease-specific supplementation	Preference-based Generic health measure Construct validity demonstrated in the general population

*FACT-G* Functional Assessment of Cancer Therapy-General, *PC* prostate cancer, *QoL* quality of life.

When collecting patient-reported outcomes (PROs) in routine clinical practice, the underlying aims include identifying and managing symptoms, monitoring disease progression, and improving communication between patient and HCP. Although valuable, these aspirational goals may not always be achievable in “real-world practice”. Clinicians often express reluctance to adopt validated assessment tools, preferring to obtain the same information by conversing with the patient in a less structured format and may lack appropriate training on the application of these instruments. For patients with PC, the length of the questionnaires and other assessment methods may pose an additional management burden. The extended consultation time required to complete these questionnaires may heighten patient anxiety and deter participation.

The use of electronic PRO (ePRO) systems should be considered to increase and improve data collection capabilities and options in clinical practice. ePRO systems may capture more complete and accurate data, improve protocol compliance, avoid data entry errors, and impose less administrative burden. Studies have reported that the use of ePRO systems resulted in survey completion rates of up to 90% in patients [87, 88].

Results from the ARAMIS, SPARTAN, and PROSPER trials have demonstrated similar trends in on-treatment QoL among patients with nmCRPC who are largely asymptomatic from their disease (Table 3). In SPARTAN, the least-squares mean change from baseline showed that HRQoL deterioration was more apparent in the placebo group than in the apalutamide group [89]. In PROSPER, a trend favoring enzalutamide was observed for all domains of the FACT-P questionnaire with the exception of physical wellbeing [90], and in ARAMIS, darolutamide significantly delayed pain progression versus placebo (40.3 vs 25.4 months; hazard ratio [HR] 0.65; 95% confidence interval [CI] 0.53–0.79; *P* < 0.001), which was maintained beyond end of study treatment [56]. In analyzing the FACT-P PC-specific subscale (FACT-P PCS), change from baseline showed no clinically meaningful difference between darolutamide and placebo [56]. Time to deterioration of European Organisation for Research and Treatment of Cancer Quality of Life Questionnaire PC module (EORTC QLQ-PR25) outcomes demonstrated statistically and clinically significant delays with darolutamide versus placebo for urinary symptoms (25.8 vs 14.8 months; HR 0.64; 95% CI 0.54–0.76; *P* < 0.01) [91].

**Table 3:** Assessment tools and patient-reported outcomes in recent clinical trials with patients with CRPC.

Trial	Assessment tools				AEs^†^	Significant assessment outcomes (drug vs PBO)^‡^
	Cognitive	QoL	Fatigue	Pain		
*Enzalutamide*						
PROSPER [50, 90] NCT02003924		FACT-P EQ-5D-5L EQ-VAS EORTC QLQ-PR25		BPI-SF	Weight decrease Decreased appetite Fatigue Asthenia Fall Mental impairment disorders	BPI-SF (pain severity): time to progression 36.83 months vs NR; HR 0.75 (95% CI 0.57–0.97; *P* = 0.028) FACT-P total score: time to deterioration in HRQoL 22.11 vs 18.43 months; HR 0.83 (95% CI 0.69–0.99; *P* = 0.037) EORTC QLQ-PR25: time to deterioration in HRQoL Bowel symptoms and function: 33.15 vs 25.89 months; HR 0.72 (95% CI 0.59–0.89; *P* = 0.0018) Hormonal treatment-related symptoms: 33.15 vs 36.83 months; HR 1.29 (95% CI 1.02–1.63; *P* = 0.035) Urinary symptoms: 36.86 vs 25.86 months; HR 0.58 (95% CI 0.46–0.72; *P* < 0.0001) EQ-VAS: time to deterioration in HRQoL 22.11 vs 14.75 months; HR 0.75 (95% CI 0.63–0.90; *P* = 0.0013)
PREVAIL [52, 105] NCT01212991		EQ-5D-3L EQ-VAS FACT-P			Fatigue Arthralgia Asthenia Weight loss Back pain Decreased appetite Fall	EQ-5D-3L (total score): average decline −0.042 vs −0.070 points (*P* < 0.0001) EQ-VAS: average decline −1.3 vs −4.4 points (*P* < 0.0001) FACT-P total score: time to deterioration in HRQoL 11.3 vs 5.6 months; HR 0.63 (95% CI 0.54–0.72; *P* < 0.001)
AFFIRM [53] NCT00974311		FACT-P		BPI-SF	Fatigue Musculoskeletal pain	BPI-SF (question no. 3; <4 vs ≥4): mean pain score HR for death 0.79 (95% CI 0.65–0.97; *P* = 0.02) FACT-P: quality of life response (10-point improvement in total score compared with baseline) 43% vs 18% (*P* < 0.001)
*Apalutamide*						
SPARTAN [54, 89] NCT01946204		FACT-P EQ-VAS EQ-5D-3L			Weight decrease Arthralgia Fatigue Fracture Fall	FACT-P: change in total score from baseline to 29 months −0.99 ± 0.98 vs −3.29 ± 1.97 EQ-VAS: change in total score from baseline to 29 months 1.44 ± 0.87 vs 0.26 ± 1.75 EQ-5D-3L (total score): similar preservation of HRQoL from baseline
*Darolutamide*						
ARAMIS [56] NCT02200614		FACT-P EQ-5D-3L EORTC QLQ-PR25		BPI-SF	Fatigue^║^	BPI-SF (pain inference): least-squares mean, time-adjusted AUC 1.1 vs 1.3 points; difference −0.2 (95% CI − 0.3 to −0.1) BPI-SF (pain severity): least-squares mean time-adjusted AUC 1.3 vs 1.4 points; difference −0.2 (95% CI − 0.3 to −0.1) FACT-P (total score): least-squares means, time-adjusted AUC 112.9 vs 111.6 points; difference 1.3; 95% CI 0.4–2.1

^†^AEs that are likely to affect the physical activity of patients and occurred with a ≥ 2% incidence in patients in the treatment group compared with the placebo group (arthralgia, asthenia, bone pain, decreased appetite, dizziness, falls, fatigue, fractures, mental impairment disorders, pain, weight loss).

^‡^Only significant results are reported in this table.

^§^None of the AEs reported met the inclusion criteria for this table.

^║^Grouped terms, for more information, see ref. [56].

*ADT* androgen deprivation therapy, *AE* adverse event, *AUC* area under the curve, *BFI-SF* Brief Fatigue Inventory Short Form, *BPI-SF* Brief Pain Inventory Short Form, *C30* 30-item core questionnaire, *CI* confidence interval, *CRPC* castration-resistant prostate cancer, *EORTC QLQ* European Organisation for the Research and Treatment of Cancer Quality of Life Questionnaire,  EQ-5D-3L EuroQol 5-Dimensions 3-Levels health questionnaire, *EQ-5D-5L* EuroQoL 5-Dimensions 5-Levels health questionnaire, *EQ-VAS* EuroQoL 5-Dimensions 5-Levels health questionnaire visual analog scale, *FACIT-Fatigue* Functional Assessment of Chronic Illness Therapy-Fatigue, *FACT-Cog* Functional Assessment of Cancer Therapy-Cognition, *FACT-*P Functional Assessment of Cancer Therapy-Prostate, *HRQoL* health-related quality of life, *PBO* placebo, *PHQ-9* Patient Health Questionnaire-9, *PR25* prostate cancer-specific 25-item questionnaire, *QoL* quality of life.

### Validated instruments used in clinical practice

The PRO questionnaires used in SPARTAN, PROSPER, and ARAMIS include some of the main PC-specific PROs adopted in clinical practice, including: the Expanded Prostate Cancer Index Composite‐26 (EPIC‐26), Expanded Prostate Cancer Index Composite‐50 (EPIC‐50), University of California‐Los Angeles Prostate Cancer Index, FACT‐P PCS, EORTC QLQ‐PR25, Prostate Cancer—Quality of Life (PC‐QoL), and Symptom Tracking and Reporting (STAR) [92].

These instruments focus on the subjective impact of physical aspects of health, with fewer items capturing the perceived impact of mental health. The EPIC-26, EPIC-50, and PC-QoL include the greatest number of items that can be labeled as “QoL”. Most of these items reflect the subjective impact of physical aspects of health, such as urinary symptoms or changes in weight. Items that capture the subjective impact of social aspects of health were less common. Although the PC-QoL included items that alluded to the impact of mental aspects of health, there was only one item about “worry” arising from the ability to sexually please a partner and categorized as an aspect of social health [92]. As these gaps in assessment domains may compromise the validity of the six instruments as well as the respective interpretation of scores and suitability for application in “real-world” patient evaluations, the true impact of PC treatment may not be conveyed via currently available HRQoL outcomes measures [92].

## Conclusions and future directions

The treatment landscape in PC is evolving rapidly, with three, second-generation ARIs available for management across the various types and stages of disease. Apalutamide, enzalutamide, and darolutamide have proven efficacious in prolonging OS, extending MFS, and demonstrating positive trends in PFS [50, 54, 56]. An important clinical consideration concerns the balance between therapeutic benefits and potential AEs that may be further complicated by DDIs with chronically administered, concomitant medications. Although second-generation ARIs demonstrate acceptable tolerability profiles in patients with CRPC, their individual drug safety and pharmacologic profiles should be considered prior to initiating combined therapy with continuous ADT. Future studies should consider the impact of chronic comorbidities on MFS, OS, and QoL. There is also a need for expert guidance on addressing common DDIs at the community practice level, especially in the absence of a clinical pharmacist.

Patient-centered communication represents a critical step toward alleviating therapeutic burden [19]. The importance of prompt and frequent updates to the history of onset and/or exacerbation of symptoms and the changes in on-treatment versus baseline activity levels should be highlighted during physician training. Close monitoring of therapeutic response over time affords HCPs the opportunity to better understand symptom onset and exacerbation in the context of an appropriately individualized treatment regimen. Healthcare providers should consider offering suggestions for preventive measures that may encourage older, comorbid patients treated for PC to participate in support groups, exercise as tolerated, and enroll in physical therapy and/or patient education programs [69–71, 73, 74]. As the financial burden of PC impacts not only the individual but also the overall healthcare system, it is important that the HCP serve as the interface between health insurers and the practice site, assuming the role of frontline patient advocate [28].

Practical approaches that enable sensitive evaluation of factors that constitute a therapeutic burden to the patient with CRPC should be incorporated into routine clinical practice to help ensure that treatment efficacy, maintenance of physical and cognitive function, and preserved QoL are considered on an individualized basis. In particular, the transition to ePRO systems should be made to help improve accuracy when collecting QoL information, as well as to alleviate the administrative burden.
