# Correction: Treating the patient and not just the cancer: therapeutic burden in prostate cancer

**DOI:** 10.1038/s41391-021-00358-9

**Published:** 2021-03-30

**Authors:** Daniel E. Spratt, Neal Shore, Oliver Sartor, Dana Rathkopf, Kara Olivier

**Affiliations:** 1 Michigan Medicine, Ann Arbor, MI USA; 2 Carolina Urologic Research Center, Atlantic Urology Clinics, Myrtle Beach, SC USA; 3 Tulane University School of Medicine, New Orleans, LA USA; 4 Memorial Sloan Kettering Cancer Center, New York, NY USA; 5 Massachusetts General Hospital Cancer Center, Boston, MA USA

**Keywords:** Cancer therapy, Prostate cancer

Correction to: *Prostate Cancer and Prostatic Diseases*

10.1038/s41391-021-00328-1

The article “Treating the patient and not just the cancer: therapeutic burden in prostate cancer”, written by Daniel E. Spratt, Neal Shore, Oliver Sartor, Dana Rathkopf and Kara Olivier, was originally published electronically on the publisher’s internet portal on 18 February 2021 without open access. With the author(s)’ decision to opt for Open Choice the copyright of the article changed on 05 March 2021 to © The Author(s) 2021 and the article is forthwith distributed under a Creative Commons Attribution 4.0 International License, which permits use, sharing, adaptation, distribution and reproduction in any medium or format, as long as you give appropriate credit to the original author(s) and the source, provide a link to the Creative Commons licence, and indicate if changes were made. The images or other third party material in this article are included in the article’s Creative Commons licence, unless indicated otherwise in a credit line to the material. If material is not included in the article’s Creative Commons licence and your intended use is not permitted by statutory regulation or exceeds the permitted use, you will need to obtain permission directly from the copyright holder. To view a copy of this licence, visit http://creativecommons.org/licenses/by/4.0/

